# Supplementary material for: The long-term dynamics of serum antibodies against SARS-CoV-2
Source: PeerJ. 2022 Dec 15;10:e14547. doi: 10.7717/peerj.14547 (PMC9760025; doi:10.7717/peerj.14547)
Supplement: Supplemental Information 2 [file peerj-10-14547-s002.docx]

**SUPPLEMENTARY MATERIAL**

**RT-qPCR and viral load estimation**

Positivity for SARS-CoV-2 infection was determined by RT-qPCR of nasopharyngeal swabs infused in 2 mL of Hanks balanced salt solution. Nucleic acids were extracted from 200 μL samples using the MagMAX Viral/Pathogen Nucleic Acid Isolation Kit (ThermoFisher Scientific), following the instructions provided by the manufacturer. Reverse transcription and cDNA amplification was carried out using the Allplex 2019-nCoV Assay kit (Seegene Inc.), incorporating the following modifications: the exogenous internal control was excluded from the reactions, only the florescent signals from RdRp and E genes were evaluated (FAM and Cal Red 610 fluorophores, respectively) and an additional human RNAse P TaqMan oligonucleotide set (VIC fluorophore; ThermoFisher Scientific) was added to the reactions as an endogenous control. RT-qPCR assays were performed on a StepOnePlus^TM^ Real Time PCR System (Applied Biosystems), following cycling specifications recommended in the kit. The cutoff Ct value for determining amplification positivity was set to 40 and patients were considered infected with SARS-CoV-2 whenever both viral genes were successfully amplified.

In order to achieve a better estimation of the viral loads, a plasmid containing cloned copies of RdRp and E genes from SARS-CoV-2, as well as the human RNAse P gene, was 10-fold serially diluted in order to correspond 10^1^ to 10^8^ gene copies per RT-qPCR reaction. Ct values obtained from these reactions were plotted in standard curves for each gene and Ct values obtained for each patient sample were interpolated into these curves to estimate RNA abundance as copy numbers per sample. As general Ct values for E gene were lower than for RdRp, and always below 30.0, this gene was used to provide the estimates of viral load (**Suppl. Fig 1A**). The detection limit of this procedure for the E gene was determined as approximately 12 RNA copies/reaction (~ 3000 copies/mL). Further normalization of copy number values took in account the endogenous RNAse P expression. Thus, all copy number values from RNAse P were first divided by its mean value, giving a normalizing factor for each sample. Then, RNA copy number calculated for the E gene from each patient were divided by the respective normalized RNAse P factor. Finally, the resulting normalized values were converted to log(10) to allow adequate graphic presentation. This normalization procedure presented more uniform and consistent estimates, as can be observed in **Supplementary Figure 1B**.

**A**

**B**

**Supplementary Figure 1: Estimates of SARS-CoV-2 viral loads, assessed by RT-qPCR of E and RdRp genes.** Data are presented as log(10) of absolute number of copies/mL (A) or normalized by endogenous RNAse P (B). Normalized E gene copy number values were used in subsequent analyses.


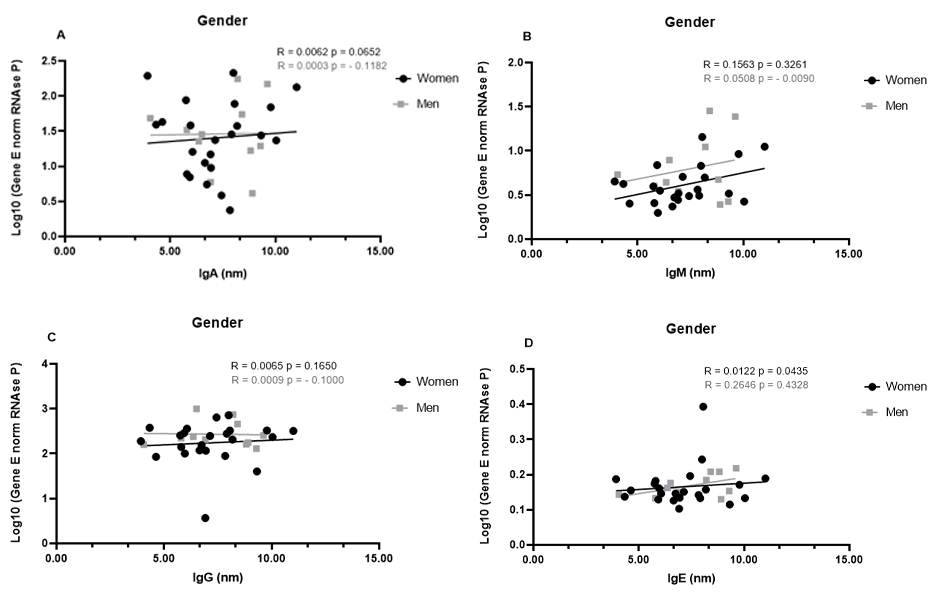


**Supplementary Figure 2: Profiles of IgA (A), IgM (B), IgG (C), and IgE (D) and viral load taking account of the gender.** A panel selected of 34 swab samples from PCR-positive mildly symptomatic SARS-CoV-2 patients was used to quantify the viral load, which was presented as log(10) and calculated according to a standard curve and number of copies/mL. The results were analyzed using Spearman’s test. The results were plotted in black for women and gray for men.


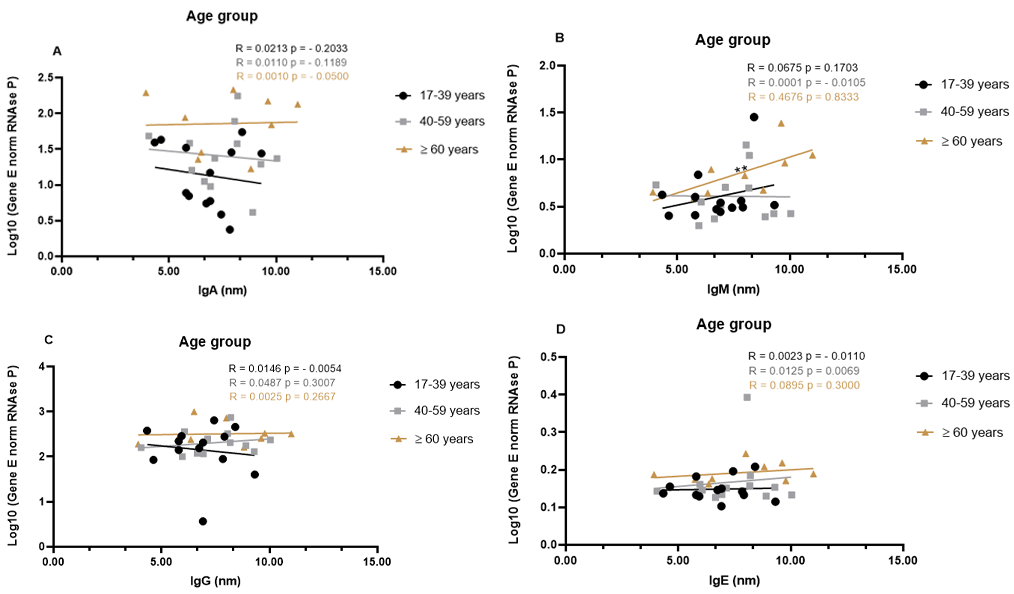


**Supplementary Figure 3: Profiles of IgA (A), IgM (B), IgG (C), and IgE (D) and viral load taking account of the age groups.** A panel selected of 34 swab samples from PCR-positive mildly symptomatic SARS-CoV-2 patients was used to quantify the viral load, which was presented as log(10) and calculated according to a standard curve and number of copies/mL. The results were analyzed using Spearman’s test. The results were plotted in black for the young adults (17-39 years), gray for the adults (40-59 years), and brown for the older adults (≥60 years old). The significance level was ** p<0.01.
